# Retention of Urine from Supposed Double Bladder

**Published:** 1830-07-01

**Authors:** 


### RETENTION OF URINE FROM SUPPOSED DOUBLE BLADDER. By M. EHRLICH.*

A man, æt. 50, who had suffered for 10 years from attacks of retention of urine, consulted M. Ehrlich on the 28th of September. He complained of being harrassed with a prolapsus of the rectum, since the appearance of which the difficulty of passing his water had increased, and now flowed only *guttatim* with insupportable pain, the bladder was full, hard, and prominent above the pubes; the anus encircled with hæmorrhoidal tumours; the cervix vesicæ swollen, but the prostate apparently healthy. The urine that was voided was so dark as to look like beer. The patient denied having ever been affected with a venereal complaint. Warm baths, demulcents, leeches to the perineum, &c. were prescribed by our author, but the patient refused to permit the introduction of the catheter. Other means were adopted, amongst the rest quinine and the tincture of the muriate of iron, but the symptoms became more severe, and on the 6th of October the catheter was introduced, with considerable difficulty and violent pain to the patient. Upwards of three pints of urine mixed with mucus were drawn off, yet still the desire of mictu-rition continued. No calculus, nor any thing like one was discovered; the extreme irritability of the individual prevented the instrument's being left in the bladder.

No more urine flowed till the 9th, when our author made many ineffectual attempts to re-introduce the instrument. On examination per anum, the bladder was felt in the left side of the pelvis, with its cervix directed towards the right. The patient being constrained to use the close-stool, made violent attempts at micturition, which ended in the expulsion of a few ounces of urine, and prolapse of the rectum to the extent of four inches. M. Ehrlich instantly reduced the latter, and succeeded in passing an instrument and abstracting more than four pints of urine. The desire of voiding more continuing, the operator suspected that some accessory pouch might exist, and succeeded in forcing the instrument, which was fourteen inches long, into a narrow passage of which he could not reach the termination, and from which about two pints of fetid urine issued. Relief was now experienced, the instrument was introduced daily with facility till the 16th, and all seemed to promise well. From this till the 25th, M. Ehrlich was prevented from attending, and his substitutes in the in-

---

* Journ. Complémentaire, No. 36. Mars, 1830.

terim had failed in carrying the catheter farther than the neck of the bladder, whilst the patient suffered from considerable hæmorrhagies from the rectum and urethra. He was now in a pitiable state, the symptoms being low and typhoid, the testicles swollen, the penis gangrenous, and the rectum prolapsed and livid. Our author punctured the bladder from the rectum, when six pints of altered bloody urine flowed out, and the prolapsus recti was reduced. The patient rallied in some degree, but the canula giving rise to great irritation, was removed, the difficulty of making water returned, and on the 28th, the operation of puncturing the rectum was repeated, after which the catheter was retained in its place for two days.

The unfavourable symptoms subsided, and on the 3d of November our author attempted to re-introduce the catheter. At first it penetrated, with some resistance, into an opening, but nothing issued, and then by manipulation it was directed into the bladder and two pints of urine obtained. On passing two fingers into the rectum, a tumour like a full bladder was felt in the left side of the pelvis, on which our author was convinced that this really was a supernumerary bladder, succeeded in getting the catheter to enter it, and evacuated three pints of urine. On injecting a bland fluid he felt this second reservoir become distended, which confirmed him in his opinion of its nature. For seven weeks it was necessary to perform the painful and difficult operation of catheterism for this unfortunate patient, but his career was drawing fast to a close. On the 22d of December he was seized with a rigor, peripneumony followed, and on the 10th of January he died.

*Sectio Cadaveris.* In the left side of the pelvis, between the rectum and ordinary bladder, was a membranous sac, equalling the latter in size, and closely united to it. The natural bladder, which we shall call the *anterior* one, was of its usual form, and in contact by its posterior surface with the unnatural, or *posterior* bladder, which was more rounded. The peritoneum was in exact contact with the posterior wall of both bladders; the anterior and external wall of the posterior bladder was united by cellular tissue to the left side of the pelvis. The right ureter terminated in the usual way; the left passed along the posterior and external surface of the second bladder, was much dilated at its point of contact with it, and passed on to the fundus of the true bladder behind the left spermatic cord and before the right. The left vesicula seminalis was closely united by cellular tissue to that of the second bladder. The prostate was only connected with the first; the veins of the plexus of the rectum and of the bladder were very much dilated.

The long muscular fibres which extend from the apex to the fundus of the bladder, were limited to the anterior one only. The posterior bladder was provided with circular and vertical muscular fasciculi, strongest at the junction of the two reservoirs. The muscular coat of the anterior bladder was three lines in thickness, so strong as to look like the columnæ carneæ of the heart, and, like them, leaving intervals between its fasciculi of fibres. The mucous membrane was not thickened. In the posterior wall of the first bladder was an aperture three lines in diameter, opening into the second. The parts around the aperture constituted the partition between the two, the parietes of which were closely and almost inseparably united.

M. Ehrlich looks on this as a satisfactory instance of a congenitally double bladder. We confess that the particulars do by no means carry conviction to our minds, but lead us to believe that the second reservoir was rather one of those exaggerated pouches from the bladder, which occasionally protrude like herniæ or staphylomata between the packets of muscular fasciculi. Many reasons, which will occur to the reflecting reader of the case, induce us to hold this opinion as being the more probable explanation of the facts. We do not readily perceive how this pouch or second bladder, be it which it may, gave rise to retention of urine. There might be a difficulty experienced in expelling its own contents, but why, or in what manner, should it operate in preventing the natural bladder, with a morbidly increased muscular power, from forcing the urine in the latter through the urethra? Surely there must have been some obstruction in the latter, and if such there was, we must look to it for the fons et origo mali!

